# Hyperinflammatory status associated with COVID-19: clinical features of a pediatric series

**DOI:** 10.1186/s42358-025-00504-0

**Published:** 2025-12-02

**Authors:** Douglas Squizatto Leite, Lucas Silva Cortes, Maria Aparecida Custódio Domingues, Claudia Saad Magalhaes, Taciana de Albuquerque Pedrosa Fernandes

**Affiliations:** 1 Rheumatology Division, Botucatu Medical School, São Paulo State University (UNESP), Botucatu, São Paulo Brazil; 2 Departament of Pathology, Botucatu Medical School, São Paulo State University (UNESP), Botucatu, São Paulo Brazil; 3 Pediatric Rheumatology Divison, Botucatu Medical School, São Paulo State University (UNESP), Botucatu, São Paulo Brazil; 4 Department of Pediatrics, Sao Paulo State University (UNESP), Rua Prof. Mario Rubens Guimaraes Montenegro SN, CEP 18618-867 UNESP Campus, Rubiao Junior, Botucatu, São Paulo Brazil

**Keywords:** COVID-19, Cytokine storm, MIS-C, Kikuchi-Fujimoto, Macrophage activation syndrome, Takayasu arteritis

## Abstract

**Objective:**

Explore clinical features, atypical manifestations and rare autoimmune complications of pediatric hyperinflammatory manifestations associated to SARS-CoV-2, observed during the COVID-19 pandemic, and the relationship of Multisystemic Inflammatory Syndrome of Childhood (MIS-C) with other manifestations, such as macrophage activation syndrome, vasculitis and vasculopathy.

**Methods:**

A protocol with detailed socio-demographic and clinical features, SARS-CoV-2 exposure and morbidity was conducted in a public tertiary hospital as part of a multicentric international protocol. Cases were selected and enrolled in a single centre from 2020 to 2022, recording all the organ and systems manifestations, standard treatment and outcome.

**Results:**

Of the 23 suspicious cases, 21 met the inclusion criteria of MIS-C. Gastrointestinal manifestations were frequent, and three out of 21 had acute abdomen, one with documented histiocytic necrotizing mesenteric lymphadenopathy (Kikuchi-Fujimoto), 3 had macrophage activation syndrome (MAS), and one case, with previous enthesitis related arthritis evolved into type V Takayasu arteritis and malignant hypertension. All patients were treated with either intravenous immunoglobulin (IVIG), high dose glucocorticoids or both, five were under intensive care treatment with respiratory and cardio-circulatory support. All had full recovery during acute phase. Description the lymphonode histopathology showed proliferation of small lymphocytes and macrophage infiltrates with microthrombi and lymph nodes germinal centers necrosis.

**Conclusion:**

Post-infectious hyperinflammatory states associated with COVID-19 manifestations may cause not only to transient inflammatory features, but also autoimmunity, vasculitis and vasculopathy manifestations that require prompt treatment.

## Background

The SARS-CoV-2 pandemic started in 2019, with severe cases of pneumonia and respiratory failure evolving into severe compromise of respiratory tract [1]. Ch]ildren and adolescents were the less affected age-group [2, 3], up to the description of a new clinical syndrome [4], that usually starts with fever, multiple organ dysfunction and high serum levels of inflammatory biomarkers [5]. The intense inflammatory process related to the viral exposure was recognized as a post-viral syndrome very similar to Kawasaki disease. The post-infectious hyperinflammatory status caused by COVID-19 is a potentially fatal condition that may evolve into cardiogenic shock and myocarditis [6, 7]. The proposed classification criteria was based on age, fever, COVID-19 exposure and multisystemic involvement, less than 21 years, fever > 38 degrees and more than 24 hours duration, two or more organ involvement including cardiovascular, renal, respiratory, hematological, gastrointestinal, dermatologic, neurologic. Confirmed exposure to COVID-19, by serology or household contact is required to confirm a suspicious case. Those criteria were revised in 2022, based on new studies and recent data, including only the most relevant signs and symptoms [8]. The relationship of COVID-19 hyperinflammatory status and autoimmune diseases, vasculopathy and vasculitis were described in other series around the world [6–12].

Given the similarities with Kawasaki disease, the treatment approach and protocols were adapted, with intravenous immunoglobulin (IVIG) as the first line treatment. Given the observed cytokine storm described in MIS-C, high dose intravenous methylprednisolone or prednisolone in variable dose regimen, as well as biologic agents, such as IL-1 and IL-6 block were reported, but it is largely depending on availability [13, 14].

This study presents a series of cases of MIS-C with atypical clinical manifestations and rare autoimmune complications during follow up.

## Method

It was a retrospective observational design study, enrolling patients with less than 21 years of age, during hospital admission, from 2020 to 2022. Cases fulfilling WHO criteria for MIS-C, with fever > 38 degrees for more than 24 hours and two or more systemic involvement; proven infection by serology, antigen or RT-PCR testing for SARS-CoV-2 or household contact with an infected person. The protocol, informed consent and assent forms were approved by the institutional ethics committee. Abnormal laboratory tests such C-reactive protein (CRP), erythrocyte sedimentation rate (ESR), fibrinogen, d-Dimer or lymphopenia indicating systemic inflammation were registered as well as the possibility of any other infection or a previous inflammatory disease ruled-out.

Macrophage activation syndrome, a life-threatening clinical manifestation associated to MIS-C was also evaluated in these cases. The diagnosis was established according to Ravelli criteria [15], namely ferritin > 684 (ng/mL) associated with two of the other features as platelets < 181 000, AST > 48, triglycerides > 156 (mg/dL) or fibrinogen < 365 (mg/dL). These criteria were originally described for Juvenile Idiopathic Arthritis (JIA), being currently used for MAS associated with other pediatric rheumatic diseases.

The demographic information, clinical manifestations, main symptoms, and laboratory tests results collected during routine hospital admission were registered in a case-report form. Data was anonymized and analyzed on excel worksheets for qualitative review and descriptive statistics. The possible associations were tested by Fischer exact test and statistical significance of 5% or correspondent p-value < 0.05.

## Results

Twenty-three suspicious cases of MIS-C were referred to the Pediatric Rheumatology Division; of those, 21 fulfilled WHO criteria for MIS-C. Mean age was 6.5 years, 57.1% female, 29% with comorbidities, 2 had seizures, 1 had previous pulmonary artery stenosis of mild degree and 3 were on follow up treatment for JIA. Out of the 21 cases included, only 18 (85.7%) underwent RT-PCR testing for SARS-CoV-2, with 12 testing negative and 6 testing positive. Serology (IgG and IgM) was performed in just 10 of the 21 patients, of whom 5 had positive results and 5 had negative results. For the remaining patients who did not undergo testing, prior COVID-19 exposure by household contacts with a positive test was confirmed. Maculopapular rash was present in 70%, and abdominal pain was the most commum gastrointestinal manifestation (60%) (Table 1).

**Table 1:** Clinical features of MIS-C patients at disease onset

Clinical Features	n (%)
Maculopapular rash	14 (70%)
Abdominal pain	13 (65%)
Vomit	10 (50%)
Conjunctivitis	8 (40%)
Tachicardia	6 (30%)
Astenia	6 (30%)
Urticaria	6 (30%)
Arthralgia	6 (30%)
Diarrhea	6 (30%)
Irritability	5 (25%)
Dispnea	5 (25%)
Hypotension	4 (20%)
Cheilitis	4 (20%)
Pharingitis	4 (20%)
Myalgia	4 (20%)
Arthritis	4 (20%)
Hepatomegaly	4 (20%)
Cough	4 (20%)
Hypoxemia (O2 Sat < 95%)	3 (15%)
Shock	3 (15%)
Muscle Weakness	3 (15%)
Lymphadenopathy	3 (15%)
Splenomegaly	3 (15%)
Myocardial dysfunction	3 (15%)
Bradicardia	2 (10%)
Myocarditis	1 (5%)
Chest pain	1 (5%)
Neutropenia	1 (5%)
Lymphopenia	8 (40%)
Trombocytopenia	3 (15%)
Hypogamaglobulinemia	3 (15%)
Hypergamaglobulinemia	2 (9.5%)
Macrophage activation syndrome	3 (14.2%)
Extremities edema	2 (9.5%)
Skin peeling	1 (4.7%)
Asseptic peritonitis	1 (4.7%)
Weight loss	1 (4.7%)
Skin ulceration	1 (4.7%)
Hepatitis	1 (4.7%)
Pneumonia	1 (4.7%)
Pleural effusion	1 (4.7%)
Asseptic meningitis	1 (4.7%)

**Fig. 1:**
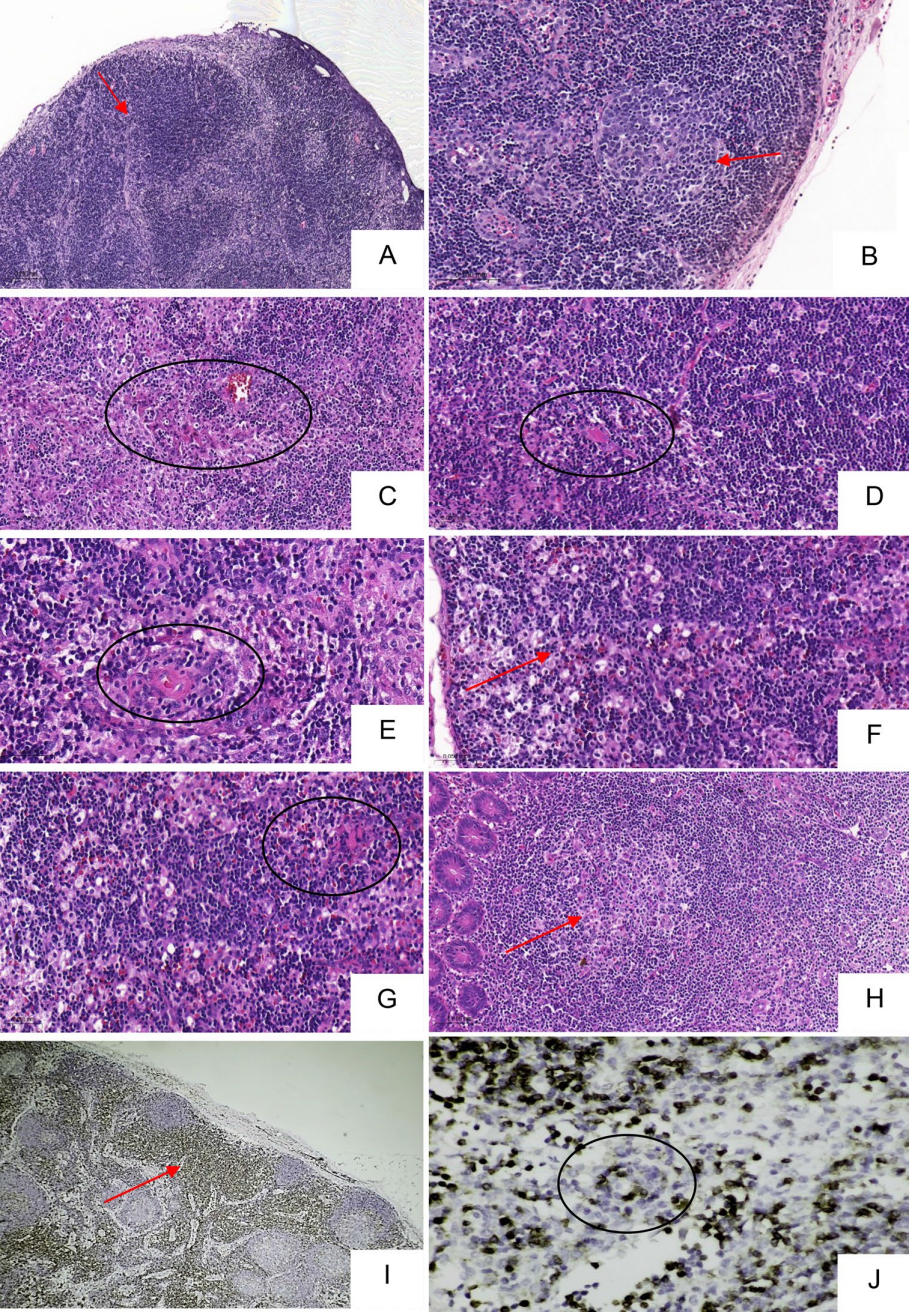
Abdominal lymph node histopathology: (**A**) Cortical region was depleted of secondary follicles that were expanded in the paracortical area HE 5X; (**B**) Germinative centres populated by small lymphocytes with karyorrhexis HE 4X; (**C**) Macrophages proliferation associated to post-capillary venules HE 24 X; (**D**) Post-capillary venules proliferation and microthrombi HE 29X; (**E**) Venule walls with fibrinoid necrosis and hyaline deposits HE 45X; (**F**) Hemophagocytosis and red blood cells out of the vessel walls HE 33X; (**G**) Intense macrophage proliferation and fibrinoid necrosis HE 33X; (**H**) Cecal Appendix histopathology: extra-vascular red blood cells, small lymphocytes proliferation HE 20X. (**I**) and (**J**) Abdominal lymph node immunohistologic positive-stain with CD3: paracortical lymph node region with expanded lymphocyte population IMH 10X; venule wall surrounded by T lymphocytes. IMH 40X. (HE: Hematoxylin-eosin stain; IMH: immunohistology stain)

Three of the cases, who had gastrointestinal manifestation, evolved into acute abdominal crisis. They had a confirmed diagnosis of MIS-C with mild respiratory symptoms, abdominal pain and positive acute phase reaction, evolving into abdominal crisis within days of the first symptoms. A pediatric surgeon evaluation in the emergency room prompted an exploratory laparotomy with suspicious of appendicitis or mesenteric lymphadenitis. The only case who had lymph nodes examined by histopathology, it was performed by conventional histology and immunohistochemistry, highlighting macrophages across the lymph nodes region layers and microtrombii typical of histiocytic necrotizing lymphadenopathy (HNL) also known as Kikuchi-Fujimoto syndrome. A detailed pictorial description of the histopathology findings is presented on Fig. 1. There was evidence of post capillary venules with high endothelial wall vessels and macrophage infiltrates, fibrinoid necrotizing tissues on germinal centers with small lymphocytes. Similar lesions were observed in cecal appendix lymphoid tissue of the same case. The other two cases who underwent laparotomy, due to appendicitis or mesenteric lymphadenitis suspicious had just lymphoid follicular hyperplasia, with no evidence of appendicitis. Coronavirus testing was not performed in any of the lymph nodes or appendix samples.

Laboratorial tests, in particular inflammatory biomarkers were: erythrocyte sedimentation rate (ESR), determined in two occasions during the hospital stay, with mean lower values (SD) 55.1 (2) mm/h and the higher 81.1 (11) mm/h; lower C-reactive protein (CRP) of 4 (0.5) mg% and higher CRP values 19.4 (0.5) mg%. Other tests were determined once during hospital admission; as mean troponin values were 10.4 (7.1) (ng/L) from 17 patients tested; mean CKMB was 20.7(13.3) (U/L). Mean pro-BNP was 6748 (pg/mL). Two of the patients had high AST and ALT with maximum AST values as 424 (U/L) (Table 2). Three patients had abnormal electrocardiogram (ECG) with tachycardia and nonspecific changes of ventricular repolarization, 5 had abnormal echocardiogram, being 2 with mild pericardial effusion. Abdominal US was carried out in 8 patients, indicating abdominal lymphadenopathy in 2 and hepatosplenomegaly in 4 cases. Abnormal chest X-ray was found in 4 patients, being 1 with “ground glass” condensation pneumonia, 1 with intersticial infiltrate, 1 with pleural effusion and 1 with lung nodules. Diagnosis of macrophage activation syndrome was done in three out of 21 patient.

**Table 2:** Laboratory parameters tested during hospital stay of 20 MIS-C cases

Laboratory Test (Units) and (Reference values)	n of tests	Mean (SD)
ESR (mm/hour) (0–20)	20	77.1 (29.8)
C-reactive protein (mg%) (<1)	20	18.65 (12.5)
Fibrinogen (mg%) (200–400)	20	447 (198.1)
D-dimer (ng/ml) (<500)	20	6527.1 (6068.8)
AST (U/L) (17–59)	20	136.6 (230.2)
ALT (U/L) (<50)	20	91.9 (126.7)
LDH (U/L) (120–246)	20	390.2 (192.4)
CK (U/L) (30–170)	20	123.9 (194.5)
Ferritin (micrL) (7–140)	20	752.7 (682.9)
Triglicerides (mg%) (<150)	14	194.4 (147.9)
C3 (mg%) (88–165)	5	125.2 (21.5)
C4 (mg%) (14–44)	5	42 (8.9)
Creatinine (mg%) (0.66–1.25)	18	0.4 (0.2)
Urea (mg%) (15–36)	18	38.4 (35.1)
Serum protein (g/dL) (6–8)	15	6.4 (1.3)
Albumin (g/dL) (3–5)	15	3.4 (0.6)
Sodium (mEq/L) (137–145)	17	129.1 (34)
Potassium (mEq/L) (3–5.1)	17	4.2 (1.2)
Troponin (ng/L) (<11)	20	84.3 (226.3)
NT-pro-BNP (pg/ml) (300–450)	15	6748 (15,291)
CKMB (U/L) (60–174)	14	348.1 (1146.9)

ESR: Erythrocyte sedimentaton rate, AST: Aspartate aminotransferase, ALT: Alanine aminotransferase, C3: Complement fraction 3, C4: Complement fraction 4, CK: Creatine kinase, CK MB: creatine kinase fraction MB, NT-Pro-BNP: N-terminal pro-B-type natriuretic peptide

MIS-C main treatment and management were: five patients needed ventilatory support; of those 2 had mechanical ventilation; 2 patients needed vasoactive-inotropic drugs and 5 patients received intravenous fluid therapy due to hypotension and shock. Intravenous immunoglobulin was prescribed for 11 cases, salicylates with doses up to 100 mg/day were prescribed to 14 cases. Six cases received IV methylprednisolone pulse with 30 mg/Kg/dose for 3 days because IVIG was not available by the time and 10 received high dose oral prednisone up to 3–4 weeks. Fourteen (66.7%) patients received nonspecific antimicrobial treatment at some point during hospital admission and 1 received acyclovir (Table 3).

**Table 3:** Management and treatment prescribed for 20 MIS-C patients

Treatment	n (%)
Salycilates (up to 100 mg/day)	14 (66.7%)
Antimicrobials	14 (66.7%)
IVIG (1–2 g/infusion)	11 (52.3%)
Prednisolone (1–2 mg/Kg/day)	10 (47.6%)
Methylprednisolone (up to 30 mg/Kg/dose) ×3	6 (28.6%)
Intravenous fluids	5 (23.8%)
Respiratory support	5 (23.8%)
Oxigen therapy	5 (20%)

IVIG: intravenous immunoglobulin

The comparison between those previously healthy and those with co-morbidities indicated no significant difference for all related variables. There was a significant statistic correlation by Fisher exact test (*p* < 0.05) of MAS and hypotension or shock status or weight loss (Table 4).

**Table 4:** Comparison of the variables associated with macrophage activation syndrome (MAS)

	MAS		p
	Yes n (%)	No (%)	
Hypotension	1 (4,8%)	3 (14.3%)	0.0030*
Tachicardia	4 (19%)	2 (9.5%)	0.1842
Bradicardia	1 (4.8%)	1 (4.8%)	0.2714
O2 Saturation < 95%	1 (4.8%)	2 (9.5%)	0.0414
Shock	2 (9.5%)	1 (4.8%)	0.4035
Astenia	5 (23.8%)	1 (4.8%)	0.6579
Irritability	3 (14.3%)	2 (9.5%)	0.1278
Weight Loss	0 (0)	1 (4.8%)	0.1429
Abnormal Chest X-Ray	2 (10%)	0 (0)	0.17
Abnormal ECG	1 (5%)	0 (0)	0.92

^*^Fisher Test

Overall, the hyperinflammatory process was transient and there was no fatal outcome or known sequels, but one case was remarked by the associated systemic vasculitis onset by the time of MIS-C presentation. A 14-years-old female with previous diagnosis of enthesitis-related-arthritis and stable JIA, treated with adalimumab and methotrexate; had COVID-19 exposure by household contact and started with fever, weight loss and seropositive COVID-19. She was treated with high dose methylprednisolone and prednisone persisting with fever, chest pain, severe abdominal pain, seizures, carotidodynia and severe hypertension due to 75% occlusion of both renal arteries. The imaging revealed type V aortic involvement and the diagnosis of Takayasu arteritis was established. She received longer cycles of high dose methylprednisolone and oral prednisone and a two-steps dilatation of renal arteries. Her treatment was switched to tocilizumab and methotrexate, reaching stable inflammatory status after 6-months.

## Discussion

This is a report from a single center series, in keeping with previosiously reported global and national series in previously healthy patients [16–19]. Unlike to precedent case-series we had a predominant frequency of females. The predominant clinical features were fever, rash in association with gastrointestinal symptoms.

The present study was an secondary exploratory analysis of the Hyper-Ped-Covid registry that described clinical presentation, management on standard treatment of COVID-19 hyperinflammatory status in 48 centers in 22 countries worldwide [20]. In the present single center report, the association of MAS and much rare cases, such as necrotizing histiocytic lymphadenitis known as Kikuchi-Fujimoto disease in mesenteric lymphnodes as well as Takayasu arteritis, were temporally associated with MIS-C hyperinflammatory status. Furthermore, it provides an innovative histopathological analysis of mesenteric lymph nodes with findings seldom described in national and international literature.

Some peculiar features were observed in our series, as the major abdominal crisis as appendicitis mimics. There are similar reports in the literature, as well as the diagnosis of other vasculitic syndromes [21, 22]. Extensive cutaneous vasculitis with microthrombi and gangrene were also reported in the literature [23].

Abdominal lymphadenopathy histopathology in one of our cases resulted in histiocytic necrotizing lymphadenopathy (HNL), also known as Kikuchi-Fujimoto syndrome, that presents with persistent fever and lymphadenopathy. The HNL is predominant in Asian children and young adults; being more often associated with peripheral lymphadenopathy and features of necrotizing vasculopathy, that may be self-limited or recurrent, with a possible autoimmune basis [24].

During the COVID-19 pandemic, cases of Kikuchi-Fujimoto findings in peripheral lymphnodes were reported as complications of SARS-CoV-2 infection in children [25, 26]. Other authors have reported a series of cases of Kikuchi-Fujimoto disease after and related to COVID-19 vaccination. Although it suggests a possible association between the vaccine and the development of the disease, a definitive causal relationship has not yet been established [27].

In our MIS-C series, high levels of inflammatory markers were inclusion criteria [24], all cases had remarkable high ESR and CRP modulated by IVIG and/or prednisolone in a few days. Heart dysfunction biomarkers have been related to severity of MIS-C and predictors of cytokine storm [28]. Myocardial dysfunction and coronary arteries dilatation and aneurisms were frequently described in the literature [29, 30]. In our series, although some abnormal myocarditis biomarkers, abnormal echocardiogram was not a frequent finding and there was no evidence of coronary arteritis or aneurisms during acute phase or during follow up. This could be related to prompt therapy with IVIG or high dose methylprednisolone.

There were three cases with evidence of macrophage activation (MAS) syndrome, needing cardio-circulatory support with intravenous fluids, vasoactive-inotropic drugs, as well as respiratory support with either non-invasive ventilation or mechanical ventilation; all cases had full recovery. MAS is a cytokine storm that occurs due to the activation of monocytes-macrophage system leading to variable degree of coagulopathy, pancytopenia, unremitting fever and lymphadenopathy, liver and central nervous system (CNS) involvement; it may co-exist with several pediatric rheumatic diseases [31] such as systemic onset juvenile idiopathic arthritis, childhood onset systemic lupus erythematosus, juvenile dermatomyositis and Kawasaki disease; usually with active underlying disease or triggered by infectious agents like Epstein-Bar virus, cytomegalovirus, herpes virus, influenzae, bacterial and parasitic infections and more recently, as part of COVID-19 hyperinflammatory states [32].

Other interesting case reported in this series was the Takayasu arteritis associated with MIS-C in the adolescent with JIA enthesitis-related arthritis as co-morbidity. Batu et al. [33] reported associated pediatric vasculitis in a systematic review of COVID-19, including 25 articles and 36 cases, with predominant IgA vasculitis followed by chilblains and ANCA-associated vasculitis. Predominant skin and renal involvement were the most typical findings. The same authors also reported a multicentric study describing a pediatric vasculitis series, referred as temporally associated with COVID-19. They identified 41 patients from 16 centers in 6 countries. The skin and gastrointestinal manifestations were the most common features. In the reported series, there was one case of a 8.5 years-old female with Takayasu arteritis, presenting with seizures and hypertension [34].

The pathogenesis of COVID-19 is complex with potentially associated immune complications. It begins in the nasopharynx, where there is abondance of ACE-2 receptor sites with interaction of SARS-CoV-2. The virus itself damages endothelium causing apoptosis, demonstrated in histopathological studies. Endothelial dysfunction and immune-thrombosis are key pathogenic mechanism in COVID-19, SARS-CoV-2 infection inducing a process of immune-thrombosis with a pathway of innate immunity triggered by pathogens and injured cells, in order to reduce the survival and spread of invading pathogens. Endothelial dysfunction was suggested as important pathophysiological event in infections by other coronavirus, which directly infected endothelial cells, leading to cellular damage and apoptosis, decreasing antithrombotic activity of the normal endothelium [35]. COVID-19 has been linked to histologically confirmed cutaneous vasculitis and Kawasaki-like vasculitis with minimal or no lung involvement and good prognosis [36]. Both small-sized and medium sized pulmonary arteriole and venule thrombosis were also reported in COVID-19. Autopsy findings of thrombosis within the walls of large pulmonary vessels with involvement of larger vessels during coronavirus disease (COVID-19), in both children and adults, due to dysfunction of their vasa vasorum and SARS-CoV-2 induced micro thrombosi would lead to hypoxic condition in the adventicia [37]. Those insights into a potential mechanism of Takayasu arteritis associated with MIS-C, a very rare event in pediatric population, point to a temporal association that suggests causality, although it is not possible rule out incidental occurrence.

The limitations of our study were a single-center small series managed during the pandemic, at a time when the knowledge and evidence-based approach were in early days. Limited resources for COVID testing, in particular the serology, and the IVIG availability, were critical. In spite of that, we were able to report severity grade and diverse fenotype expressions and rare autoimmune manifestations related to viral infection.

In conclusion, MIS-C has a wide spectrum of clinical features and potentially life-threatening complications, including rare manifestations such as necrotizing histiocytic lymphadenopathy, macrophage activation syndrome and large vessel vasculitis. Awareness, prompt diagnosis and timely treatment start was critical for the outcome in all those manifestations.

## Data Availability

All data and materials are available from the first author files and the instituition´s repository.

## Abbreviations

COVID-19: Coronavirus disease
SARS-CoV-2: Severe acute respiratory syndrome coronavirus 2
MIS-C: Multisystemic Inflammatory Syndrome of Childhood
HNL: Histiocytic necrotizing lymphadenopathy
MAS: Macrophage activation syndrome
IVIG: Intravenous immunoglobulin
WHO: World Health Organization
CRP: C-reactive protein
ESR: Erythrocyte sedimentation rate
JIA: Juvenile Idiopathic Arthritis
AST: Aspartate aminotransferase
ALT: Alanine aminotransferase
ECG: Electrocardiogram
US: Ultrasound
IV: Intravenous
CNS: Central nervous system
IgA: Immunoglobulin A
ANCA: Anti-neutrophil cytoplasmic antibody
ACE-2: Angiotensin-converting enzyme 2
